# Clinical and economic benefits of image-guided system in functional endoscopicsinus surgery: a retrospective chart review study in China

**DOI:** 10.1186/s12962-023-00414-2

**Published:** 2023-01-12

**Authors:** Zaixing Wang, Chunling Liu, Baoying Tan, Guoqing Sun, Poucheok Pang, Kangchen lv, Hui Xie, Xiong Ou, Xianhai Zeng, Jianwei Xuan

**Affiliations:** 1 Department of Otolaryngology, Shenzhen Longgang E.N.T Hospital & Shenzhen Key Laboratory of E.N.T, Institute of E.N.T, Shenzhen, China; 2 The Second Affiliated Hospital of Shenzhen University (People’s Hospital of Shenzhen Baoan District), Shenzhen, China; 3 School of Pharmacy, Health Economic Research Institute, Sun Yat-Sen University, Guangzhou, China; 4 Medtronic (Shanghai) Co. LTD, Shanghai, China

**Keywords:** Image-guided system, Functional endoscopic sinus surgery, Clinical and economic benefits

## Abstract

**Background:**

Image-guided system (IGS) has been gradually applied in the field of rhinology, making functional endoscopic sinus surgery (FESS) a truly minimally invasive and precise surgery. This study was based on real-world data from China hospitals and aimed to evaluate the clinical and economic benefits of the IGS navigation system in FESS.

**Methods:**

This was a two-center retrospective chart review of patients with chronic rhinosinusitis who underwent FESS, including open frontal sinus between July 1, 2018 and December 31, 2019 in China. The intervention group consisted of 100 patients who underwent FESS with the IGS navigation system (IGS group), and the control group consisted of 100 patients who underwent conventional FESS (Non-IGS group). Data were collected from surgical notes and hospital medical records. The primary endpoints for clinical effectiveness and safety were avoid rehospitalization due to bleeding, avoid reoperation due to bleeding, and avoid reoperation due to recurrence.

**Results:**

There were no cases of rehospitalization due to bleeding, reoperation due to bleeding, and reoperation due to recurrence in the IGS group, with an avoidance rate of 100%. In the non-IGS group, there were four cases of rehospitalization and reoperation due to bleeding, with an avoidance rate 96.00% (P = 0.121). No cases of reoperation due to recurrence were in the non-IGS group. The total hospitalization cost was 17,391.51 CNY in the IGS group and 17,742.41 CNY in the non-IGS group per patient, with no statistical difference between the two groups (P = 0.715). Compared with the non-IGS group, the IGS group had an overall cost saving of 350.90 CNY per patient. Although the procedure-related medical costs of IGS group were increased by 1,286.12 CNY compared with the non-IGS group, this was more than offset by other costs.

**Conclusion:**

The results of the study indicated that the IGS may avoid occurrence of rehospitalization and reoperation due to postoperative bleeding. Although the use of navigation technology increased the cost of surgery, its clinical effectiveness brought other medical cost savings, resulting in no significant difference in the overall cost of navigation surgery compared to conventional surgery. The IGS should be considered cost-effectiveness in the treatment of FESS.

**Supplementary Information:**

The online version contains supplementary material available at 10.1186/s12962-023-00414-2.

## Background

Chronic sinusitis is a chronic inflammatory disease of the mucous membrane of the sinuses that is purulent [1, 2]. The self-reported prevalence of chronic sinusitis in China was 2.2% in 2017, with approximately 31 million people suffering from this condition [3]. Chronic sinusitis not only imposes a financial burden on patients, but also causes a large consumption of medical resources and even loss of productivity to society. The average annual number of visits for patients with chronic sinusitis in 2018 was 4.5 in China with an average of 11.7 days of sick leave [4–6]. In the United States, the average annual cost for a typical chronic sinusitis patient is $1,983, and this disease adds about $14.4 billion to the healthcare cost each year [5]. Clinically, chronic sinusitis exhibits a range of symptoms, such as persistent nasal congestion, increased occlusive nasal sounds, loss of smell, and snoring during sleep, which may seriously affects the quality of life of patients [7]. A study using the MOS 36-item short-form health survey (SF-36) to investigate the quality of life of patients with chronic sinusitis showed that patients with chronic sinusitis had significantly lower scores in the seven dimensions of physical role, body pain, general health, vitality, social functioning, emotional role, and psychological health compared to the healthy population [8].

The current guidelines for the diagnosis and treatment of chronic sinusitis in China (2018), recommend that the first-line treatment for chronic sinusitis is pharmacotherapy, with glucocorticoids for patients with chronic sinusitis, and then mucolytic pro-discharge agents, decongestants or nasal rinses as an adjuvant therapy taking into consideration of each patient’s condition [1]. When drug therapy is not effective, functional endoscopic sinus surgery (FESS) is the main treatment. FESS can remove irreversibly diseased tissue from the sinuses, preserve the intact sinus mucosa, reestablish sinus ventilation, reduce the inflammatory response, and restore mucosal glandular and ciliary clearance [1]. Because the sinuses are adjacent to important sites such as the nasolacrimal duct, orbit, optic nerve, cavernous sinus, internal carotid artery, skull base, and slope, there is a risk of serious postoperative complications, which may include nasolacrimal duct and lacrimal sac injury, orbital wall injury, visual impairment, cerebrospinal fluid rhinorrhea, and hemorrhagic complications [9]. One study reported a 1.00% incidence of complications associated with FESS (including 0.17% for cerebrospinal fluid nasal leakage, 0.07% for orbital injury, and 0.76% for transfusion events) [10].

With continuous development of minimally invasive technology, image-guided system (IGS) has been gradually applied in the field of rhinology, making FESS a truly minimally invasive and precise surgery, which can help the operator locate anatomical landmarks with great precision during FESS and improve surgery success rate with reduced risk of complications. Fusion^®^ ENT special electromagnetic navigation system (Medtronic, USA) is a special navigation system designed for ENT head and neck surgery, which can reconstruct patient's preoperative axial CT scan image in three dimensions. The reconstructed images are highly accurate and free of infrared occlusion problems. The quick registration of the navigation system is easy and fast to use, and it is connected with the power system to provide real-time images. The image navigation makes FESS truly minimally invasive and precise, with short preoperative preparation time and high registration accuracy, which can improve the safety of surgery and reduce the occurrence of complications. A clinical study in China of 60 patients undergoing complex FESS assisted by Fusion navigation showed no intracranial or orbital complications, and its clinical value was well demonstrated [11].

Adoption of innovative medical devices should be evaluated on multiple dimensions of value attributes, including clinical, economic, patients, and social value. Among them, safety and effectiveness are the intrinsic properties for innovative technologies to be clinically applied. Some studies have shown that the use of IGS for FESS may cause local skin irritation but its benefits to patients outweigh the harms, and IGS may reduce the occurrence of secondary sinus surgery [12]. The results of a META study showed that, compared with conventional FESS, IGS-assisted FESS reduced the incidence of major complications of FESS, including reoperation due to postoperative bleeding (RR 0.48; 95% CI 0.28–0.82; P = 0.007) and overall complications (RR = 0.66; 95% CI 0.47–0.94; P 0.02) [13]. A cost study showed that the cost of IGS was increased by only 6.7% compared with conventional FESS and the intangible clinical benefits of the navigation system may outweigh its increased cost [14]. In the context of value-based healthcare, innovative technologies should reflect their cost-effectiveness compared with existing technologies. This study was based on real-world data from China hospitals and aimed to evaluate the cost effectiveness of the IGS navigation system in FESS.

## Methods

### Study design

We conducted a retrospective chart review of patients with chronic rhinosinusitis who met the inclusion and exclusion criteria from two tertiary care hospitals in Shenzhen, Otolaryngology Hospital of Longgang District (Hospital A), Shenzhen Bao'an People's Hospital (Hospital B). IGS was only available in hospital A, which became available in 2018. Two qualified surgeons who contributed patients to this study respectively, whose title were associate chief physician. The intervention group consisted of 100 consecutive eligible patients who underwent FESS with the IGS navigation system (IGS group), and the control group consisted of 100 consecutive eligible patients who underwent conventional FESS (Non-IGS group). The study protocol was approved by the respective hospital ethics committee of two principal investigators and written informed consent was obtained from all patients.

### Inclusion and exclusion

Patients who met all of the following inclusion criteria were included: (1) underwent a FESS, including open frontal sinus, between July 1, 2018 and December 31, 2019; and (2) were greater than or equal to 18 years old and less than 75 years old.

Patients with any of the following exclusion criteria were excluded: (1) involved in skull base surgery; (2) malignant tumors that required resection in the surgical plan or were confirmed postoperatively; or (3) patients with bleeding tendencies (e.g., long-term use of anticoagulant medications, coagulopathy, bleeding events within the last month) that, in the opinion of the investigators, could affect assessment of clinical endpoints.

### Outcome

Data were collected from surgical notes and hospital medical records. Baseline characteristics included age, gender, site of sinus removed during FESS, number of sinuses removed (unilateral or bilateral), previous surgical history, and disease history.

The primary endpoints for clinical effectiveness and safety were the avoid rehospitalization due to bleeding, avoid reoperation due to bleeding, and avoid reoperation due to recurrence. The secondary endpoints were length of stay, length of stay due to bleeding, length of stay after procedure, number of blood transfusions, and number of serious adverse events. Serious adverse events associated with surgery included perioperative bleeding, orbital injury (sieve bone paper sample loss, intra-orbital hematoma, retrobulbar hemorrhage, nasolacrimal duct injury, or optic nerve loss), intracranial injury (cerebrospinal fluid leakage or intracranial infection), and bleeding after discharge from hospital.

The total inpatient costs included expenses associated with medical service, diagnosis, procedure, procedure-related consumables, medications, hospitalization-related treatment, nursing, and rehabilitation. Medical service costs entailed consultation and bed costs. Diagnostic costs included pathology and laboratory diagnosis, and imaging studies. Procedure-related costs were the costs of treatment operations during surgery. Procedure-related consumables costs were the disposable medical materials costs during surgery including the amortization costs and the cost of maintenance. Hospitalization-related treatment operation costs were the injection, debridement, dressings change, catheterization, and oxygen costs.

### Statistical analysis

R statistical analysis software (*R Studio Version 1.3.959*) was used to perform descriptive statistical analysis and univariate analysis. The results of descriptive statistics were reported as mean, standard deviation, median, and upper and lower quartiles for continuous variables, and as frequencies and percentages for categorical variables. Inferential statistics were selected based on the specifics of the data distribution with different hypothesis testing methods, using Student's t-test for continuous variables and chi-square test or Fisher's exact test for categorical variables. All tests were two-sided, and P < 0.05 was considered statistically significant.

## Results

In the study population, patients undergoing FESS were approximately 41 years old, with more male patients than female patients. In the IGS group and non-IGS group, 65% and 74% of patients had surgery involving the sphenoid sinus, respectively, which was not statistically different. Significantly more patients in the IGS group had bilateral maxillary sinuses involvement compared to non-IGS group (79% vs. 67%; P = 0.044) and other baseline characteristics of the two groups were similar and comparable with regard to age, gender, previous surgical history, and disease history (all P > 0.05) (Table 1).

**Table 1:** Baseline characteristics for patients with functional endoscopic sinus surgery

Variables	Overall patients		
	IGS	Non-IGS	P-value
Number of patients	100	100	–
Age			
Mean (SD)	41.25 (11.24)	41.50 (12.58)	0.882
Median [IQR]	33 [40,50]	32 [42,50]	0.894
Gender, n (%)			
Male (%)	65 (65.00)	74 (74.00)	0.219
Female (%)	35 (35.00)	26 (26.00)	
Number of frontal sinuses, n (%)			
1 (Unilateral)	30 (30.00)	45 (45.00)	0.041
2 (Bilateral)	70 (70.00)	55 (55.00)	
Number of sphenoid sinuses, n (%)			
0	62 (62.00)	54 (54.00)	0.497
1 (Unilateral)	11 (11.00)	12 (12.00)	
2 (Bilateral)	27 (27.00)	34 (34.00)	
Number of ethmoid sinuses, n (%)			
0	0 (0.00)	2 (2.00)	0.141
1 (Unilateral)	22 (22.00)	30 (30.00)	
2 (Bilateral)	78 (78.00)	68 (68.00)	
Number of maxillary sinuses, n (%)			
0	0 (0.00)	4(4.00)	0.044
1 (Unilateral)	21 (21.00)	29 (29.00)	
2 (Bilateral)	79 (79.00)	67 (67.00)	
History of previous surgery, n (%)			
Sinus related surgery	13 (13.00)	7 (7.00)	0.239
Functional endoscopic sinus surgery	1 (1.00)	2 (2.00)	1.000
History of disease, n (%)			
Hypertension	5 (5.00)	13 (13.00)	0.084
Diabetes	2 (2.00)	3 (3.00)	1.000
Kidney disease	1 (1.00)	1 (1.00)	1.000
Hepatobiliary disease	4 (4.00)	4 (4.00)	1.000
Gastrointestinal disease	1 (1.00)	6 (6.00)	0.124
Lung disease	7 (7.00)	3 (3.00)	0.330

There were no cases of rehospitalization due to bleeding, reoperation due to bleeding, and reoperation due to recurrence in the IGS group, with an avoidance rate of 100%. In the non-IGS group, there were four cases of rehospitalization and reoperation due to bleeding, with an avoidance rate 96.00% (P = 0.121). No cases of reoperation due to recurrence were in the non-IGS group (Table 2).

**Table 2:** Efficacy and safety outcomes for patients with functional endoscopic sinus surgery

Variables	Overall patients		
	IGS	Non-IGS	P-value
Number of patients	100	100	–
Avoid rehospitalization due to bleeding, n (%)	100 (100.00)	96 (96.00)	0.121
Avoid reoperation due to bleeding, n (%)	100 (100.00)	96 (96.00)	0.121
Avoid reoperation due to recurrence, n (%)	100 (100.00)	100 (100.00)	–
Procedure time, min			
Mean (SD)	99.35 (42.04)	104.13 (38.12)	0.401
Median [IQR]	100.00 [75.00, 125.00]	100.00 [74.25, 130.00]	0.380
Length of stay, day			
Mean (SD)	5.95 (1.02)	5.43 (2.21)	0.034
Median [IQR]	6.00 [5.00, 6.00]	5.00 [4.00, 6.00]	< 0.01
Length of stay after procedure, day			
Mean (SD)	3.88 (0.81)	3.05 (0.89)	< 0.001
Median [IQR]	4.00 [3.00, 4.00]	3.00 [2.00, 4.00]	< 0.001
Length of stay due to bleeding, day	n = 0	n = 4	
Mean (SD)	–	4.00 (2.58)	–
Median [IQR]	–	4.00 [2.00, 6.00]	
Blood transfusion, n (%)	0 (0.00)	0 (0.00)	–
Serious adverse event, n (%)	0 (0.00)	4 (4.00)	0.121
Perioperative bleeding, n (%)	0 (0.00)	0 (0.00)	–
Orbital injury, n (%)	0 (0.00)	0 (0.00)	–
Intracranial injury, N (%)	0 (0.00)	0 (0.00)	–
Bleeding after discharge from hospital, n (%)	0 (0.00)	4 (4.00)	0.121

There was no statistical difference in procedure time between the IGS group and the non-IGS group (P = 0.401). The length of stay and the length of day after procedure were longer in the IGS group than in the non-IGS group (P < 0.05). No blood transfusion events, perioperative bleeding events, orbital injury events, or intracranial injury events occurred in either group (Table 2).

The total hospitalization cost including follow-up inpatient costs due to bleeding was 17,391.51 CNY in the IGS group and 17,742.41 CNY in the non-IGS group per patient, with no statistical difference between the two groups (P = 0.715). Compared with the non-IGS group, the IGS group had an overall cost saving of 350.90 CNY per patient. Although the procedure-related medical costs were increased by 1286.12 CNY compared with the non-IGS group, this was offset by other cost components, including the diagnosis costs, procedure-related consumables, drugs, hospitalization-related treatment, rehabilitation, and use of antimicrobials (Table 3). In the two groups of patients available for follow-up (IGS group: n = 91; non-IGS group: n = 99), the 12-Month follow-up outpatient costs due to regular visits after FESS were comparable and not statistically different (IGS group: 1362.15 CNY; Non-IGS group: 1318.52 CNY; P = 0.744). The average 12-Month follow-up inpatient cost due to bleeding was 5,107.03 CNY for the four patients who had rehospitalization due to bleeding in the non-IGS group.

**Table 3:** Healthcare costs for all patients with a functional endoscopic sinus surgery

Variables	Overall patients		
	IGS	Non-IGS	P-value
Number of patients	100	100	
Total costs (Including follow-up inpatient costs due to bleeding)			
Mean (SD)	17,391.51 (2,397.70)	17,742.41 (3498.82)	0.204
Median [IQR]	17,447.92 [15,226.44, 19,103.97]	18,049.26 [15,427.10, 19,851.24]	0.298
Medical service costs			
Mean (SD)	421.86 (120.72)	453.44 (237.24)	0.237
Median [IQR]	360.00 [360.00, 488.80]	385.00 [300.00, 539.20]	0.913
Diagnosis costs			
Mean (SD)	1,987.98 (548.64)	2,256.84 (1,047.65)	0.024
Median [IQR]	1,979.50 [1,601.50, 2,370.20]	2,122.00 [1,814.95, 2,427.80]	0.075
Procedure-related medical costs			
Mean (SD)	11,446.99 (1,806.33)	10,160.87 (2,379.05)	< 0.001
Median [IQR]	11,645.00 [9,973.28, 12,626.07]	10,641.50 [8,430, 12,028.70]	< 0.001
Procedure-related consumables costs			
Mean (SD)	1,588.19 (500.08)	1,812.30 (693.80)	0.009
Median [IQR]	1,497.96 [1,263.63, 1,810.83]	1,742.22 [1,495.88, 2,057.89]	0.001
Drug costs			
Mean (SD)	1,580.26 (415.61)	2,344.27 (573.31)	< 0.001
Median [IQR]	1,574.27 [1,279.59, 1,861.63]	2,324.01 [2,009.34, 2,685.82]	< 0.001
Antibacterial drugs in the drug costs			
Mean (SD)	198.85 (366.30)	487.57 (193.02)	< 0.001
Median [IQR]	49.98 [49.98, 49.98]	465.24 [349.97, 591.44]	< 0.001
Hospitalization-related treatment costs			
Mean (SD)	190.48 (44.99)	331.49 (165.04)	< 0.001
Median [IQR]	188.50 [173.88, 207.13]	260.92 [202.85, 473.98]	< 0.001
Nursing costs			
Mean (SD)	175.75 (28.53)	141.12 (52.03)	< 0.001
Median [IQR]	176.00 [160.00, 189.00]	128.50 [111.00, 159.00]	< 0.001
Rehabilitation costs			
Mean (SD)	0.00 (0.00)	37.80 (29.11)	< 0.001
Median [IQR]	0.00 [0.00, 0.00]	60.00 [0.00, 60.00]	< 0.001
12-Month follow-up inpatient costs due to bleeding (n =)	n = 0	n = 4	–
Mean (SD)	–	5,107.03 (2,288.41)	–
Median [IQR]	–	4,236.15 [3,664.19, 5,679.00]	

A subgroup analysis of patients whose surgery involving the sphenoid sinuses was subsequently conducted. There was no statistical difference in baseline characteristics between the IGS group and the non-IGS group (P > 0.05) (Additional file 1: Table S1), and there were two cases of rehospitalization due to bleeding. The total hospitalization cost was 18,764.23 CNY in the IGS group and 19,624.92 CNY in the non-IGS group per patient, which was not statistically different (P = 0.076). Although the IGS group had an increase of 833.11 CNY in procedure-related medical costs compared with the non-IGS group, this was more than offset by other cost items, i.e., drugs, hospitalization-related treatment, rehabilitation, and use of antimicrobials, resulting in an overall cost savings of 878.69 CNY per patient. The average 12-Month follow-up inpatient cost due to bleeding was 6077.32 CNY for the two patients who had rehospitalization due to bleeding in the non-IGS group (Additional file 1: Table S4).

## Discussion

To our knowledge, our study was the first cost-effectiveness analysis related to IGS-assisted FESS in patients with chronic rhinosinusitis in China in real world settings. The results showed that the navigation technique may avoid the incidence of rehospitalization for postoperative bleeding in real world settings. In the present study, we found that that the avoid rehospitalization due to bleeding and avoid reoperation due to bleeding were significantly higher in the IGS group than in the non-IGS group (100.00% vs 96.00%; P = 0.121) and the four cases of rehospitalization and reoperation due to postoperative bleeding were shown in Table 4. We believe the improved clinical outcome was associated with the application of the IGS for its ability to map out anatomical structures with great precision, especially in patients with anatomical variants, which had a significant guiding effect on surgery. Other studies have shown higher incidences of complications in both groups. This may be due to the use of different IGS systems and the disease severity of the study population. A meta-analysis [13] indicated that major complications were more common in the non-IGS group (IGS group: 1.3%; non-IGS group: 3.3%) and total complications were greater in the non-IGS group (IGS group: 3.9%; non-IGS group: 6.3%). All other outcomes including periorbital injuries, intracranial injuries, and major hemorrhage did not reach significance. Pooled results did not show any significant benefit of IGS over non-IGS with regard to need of additional revision surgery (IGS group: 7.3%, non-IGS group: 9.4%). Fried et al. [15] showed a statistically significant benefit of the IGS group over the non-IGS group. The major complication rate in the IGS group was 1% due to orbital entry. However, in the non-IGS group, the major complication rate was 11.1%, including 4.8% for orbital entries, 3.2% for operation halted due to bleeding, 1.6% for dual dehiscence, and 1.6% for procedures halted due to patient hypotension. And the repeat surgery rate was 1.6% in the IGS group and 4.8% in the non-IGS group within 3 months. Interestingly, Mueller et al. [16] found a trend towards a slightly lower major complication rate in the non-computer-assisted group although this difference was not statistically significant (6.0%, compared with 6.5% in the computer-assisted group). It was notable that complications in the computer-assisted group were limited to bleeding, while orbital complications and skull base injury only occurred in the non-computer-assisted group. In addition, patients in the computer-assisted group generally showed more extensive disease on pre-operative CT scans. The revision surgery rate was 9.2% in the computer-assisted group and 10.7% in the non-computer-assisted group.

**Table 4:** Case reports of rehospitalization and reoperation due to postoperative bleeding

Patients	Rebleeding after FESS	Intraoperative bleeding
Patient A	5 days after surgery and left nasal bleeding for one day	20 ml
Patient B	One week after surgery and nose bleeding for 10 h	50 ml
Patient C	2 weeks after surgery and recurrent right nasal bleeding for 4 days	5 ml
Patient D	8 days after surgery and recurrent left nasal bleeding for 4 h	2 ml

In our study, it was noted that the IGS group consisted of a more complex patient population with bilateral frontal sinuses. Nevertheless, despite the increased case complexity, the IGS group had no cases of complication. On the other hand, use of IGS in the patient depended on whether the treating surgeon was appropriately trained in the IGS system and it was evident that a single surgeon’s learning curve may affect the outcome of surgery. There were no cases of reoperation rate due to recurrence in either the IGS group or the non-IGS group, which may be related to the sample size selected and the length of follow-up for our study.

The cost results in our study showed that although the cost of using the navigation system was increased in the IGS group compared to the non-IGS group, it was totally offset by other cost components, resulting in similar total cost between the two groups. The IGS group saved the costs from incurring complications due to the avoidance of postoperative bleeding and rehospitalization events. In clinical practice, the surgical cost was mainly influenced by the number of diseased sinuses and was not related to the use of navigation systems. In our subgroup analysis, patients with sphenoid sinus were more difficult to operate on and more severely ill than patients without sphenoid sinus. It is worth noting that in the sphenoid sinus group, the advantage of the navigation technique was more discernible as shown in a previous study, which concludes that application of IGS is beneficial in revision sphenoid sinus surgery [17].

Economic studies of the use of IGS for FESS were limited and published studies in general concluded that navigation systems, although expensive, were cost-effective. A cost study showed that the overall cost was 6.7% higher in the IGS group than in the non-IGS group, and the increased cost in the IGS group may be related to the positioners and suction devices required for the patients and the need for additional CT scans in some patients [14]. Another study investigating the perception of the IGS use for sinus surgery among otolaryngologists in the United States found that most respondents believed that the use of IGS in frontal sinus surgery may lead to greater safety, but that ease of use, complexity of technical setup, local reimbursement, and cost of purchase may limit routine clinical use, so that expanded use may depend on ease of use, reimbursement policies, and affordability [18].

Potential limitations should be taken into consideration when interpreting the findings of this study. Firstly, this study focused on conventional surgery in which the frontal sinus must be respected, excluding surgery with combined serious diseases, and did not consider patients with more complex surgery and malignant tumors. The effect of navigation technology applied to patients with complex conditions deserves further exploration. Secondly, the limited number of hospitals included may have introduced patient selection bias and the severe patients may choose to undergo surgery at hospitals with navigation technology available in clinical practice. Thirdly, as our study was descriptive in nature, the sample size was determined based on feasibility and convenience and is relatively small. Finally, no sensitivity analysis was performed on costs in this study, as costs may be affected by other factors. Despite the above limitations, none of them would systemically bias the findings in favor of the IGS group. Future studies with large multicenter samples could be conducted. The long-term clinical benefits could be collected by following up with the patients over time, focusing not only on the incidence of perioperative complications but also on the recurrence of sinusitis. Patient-reported outcomes could also be collected through a disease-specific quality of life questionnaire to reflect the impact of the navigation system on patients' quality of life. Considering that the operators of IGS are healthcare professionals, the value of the IGS should also be evaluated in terms of healthcare use experience to provide more substantial clinical promotion of the image navigation system in the field of ENT.

## Conclusion

Navigation technology is used as an adjunctive treatment tool in functional endoscopic sinus surgery. The results of the study indicated that the IGS may avoid occurrence of rehospitalization and reoperation due to postoperative bleeding. Although the use of navigation technology increased the cost of surgery, its clinical effectiveness brought other medical cost savings, resulting in no significant difference in the overall cost of navigation surgery compared to conventional surgery. The IGS should be considered cost-effectiveness in the treatment of FESS.

## Supplementary Information

**Additional file 1: Table S1.** Baseline characteristics for patients with functional endoscopic sinus surgery. **Table S2.** Efficacy and safety outcomes for patients with functional endoscopic sinus surgery. **Table S3.** Healthcare costs for all patients with functional endoscopic sinus surgery. **Table S4.** Healthcare costs for subgroup patients with functional endoscopic sinus surgery.

## Data Availability

All related data were displayed in the manuscript. Further information regarding the data can be obtained by contacting the corresponding authors.

## Abbreviations

IGS: Image-guided system
FESS: Functional endoscopic sinus surgery
SF-36: The MOS 36-item short-form health survey
