# Supplementary material for: Clinical and economic benefits of image-guided system in functional endoscopicsinus surgery: a retrospective chart review study in China
Source: Cost Eff Resour Alloc. 2023 Jan 12;21:1. doi: 10.1186/s12962-023-00414-2 (PMC9837985; doi:10.1186/s12962-023-00414-2)
Supplement: Supplementary file 1 — Additional file 1: Table S1. Baseline characteristics for patients with functional endoscopic sinus surgery. Table S2. Efficacy and safety outcomes for patients with functional endoscopic sinus surgery. Table S3. Healthcare costs for all patients with functional endoscopic sinus surgery. Table S4. Healthcare costs for subgroup patients with functional endoscopic sinus surgery. [file 12962_2023_414_MOESM1_ESM.docx]

## Additional file information

Additional file 1.

**Additional file 1: Table 1. Baseline characteristics for patients with functional endoscopic sinus surgery**

| **Variables** | **Overall patients** | | | **Surgery for patients with sphenoid sinus** | | | **Surgery for patients without sphenoid sinus** | | |
| --- | --- | --- | --- | --- | --- | --- | --- | --- | --- |
|  | **IGS** | **Non-IGS** | **P-value** | **IGS** | **Non-IGS** | **P-value** | **IGS** | **Non-IGS** | **P-value** |
| **Number of patients** | 100 | 100 | - | 38 | 46 | - | 62 | 54 | - |
| **Age** | | | | | | | | | |
| Mean (SD) | 41.25 (11.24) | 41.50 (12.58) | 0.882 | 41.24 (11.23) | 40.98 (11.88) | 0.919 | 39 (62.90) | 36 (66.67) | 0.819 |
| Median [IQR] | 33 [40,50] | 32 [42,50] | 0.894 | 38 [34,39] | 33 [40,48] | 0.801 | 33 [40,51] | 30 [45,51] | 0.67 |
| **Gender, n (%)** | | | | | | | | | |
| Male (%) | 65 (65.00) | 74 (74.00) | 0.219 | 26 (68.42) | 38 (82.61) | 0.207 | 39 (62.90) | 36 (66.67) | 0.819 |
| Female (%) | 35 (35.00) | 26 (26.00) |  | 12 (31.58) | 8 (17.39) |  | 23 (37.10) | 18 (33.33) |  |
| **Number of frontal sinuses, n (%)** | | | | | | | | | |
| 1 (Unilateral) | 30 (30.00) | 45 (45.00) | 0.041 | 3 (3.00) | 11 (11.00) | 0.076 | 27 (27.00) | 34 (34.00) | 0.042 |
| 2 (Bilateral) | 70 (70.00) | 55 (55.00) |  | 35 (35.00) | 35 (35.00) |  | 35 (35.00) | 20 (20.00) |  |
| **Number of sphenoid sinuses, n (%)** | | | | | | | | | |
| 0 | 62 (62.00) | 54 (54.00) | 0.497 | 0 (0.00) | 0 (0.00) | 0.963 | 62 (100.00) | 54 (100.00) | NA |
| 1 (Unilateral) | 11 (11.00) | 12 (12.00) |  | 11 (28.95) | 12 (26.09) |  | 0 (0.00) | 0 (0.00) |  |
| 2 (Bilateral) | 27 (27.00) | 34 (34.00) |  | 27 (71.05) | 34 (73.91) |  | 0 (0.00) | 0 (0.00) |  |
| **Number of ethmoid sinuses, n (%)** | | | | | | | | | |
| 0 | 0 (0.00) | 2 (2.00) | 0.141 | 0 (0.00) | 0 (0.00) | 0.488 | 0 (0.00) | 2 (3.70) | 0.102 |
| 1 (Unilateral) | 22 (22.00) | 30 (30.00) |  | 3 (7.89) | 7 (15.22) |  | 19 (30.65) | 23 (42.59) |  |
| 2 (Bilateral) | 78 (78.00) | 68 (68.00) |  | 35 (92.11) | 39 (84.78) |  | 43 (69.35) | 29 (53.70) |  |
| **Number of maxillary sinuses, n (%)** | | | | | | | | | |
| 0 | 0 (0.00) | 4(4.00) | 0.044 | 0 (0.00) | 0 (0.00) | 0.488 | 0 (0.00) | 4 (7.41) | 0.024 |
| 1 (Unilateral) | 21 (21.00) | 29 (29.00) |  | 3 (7.89) | 7 (15.22) |  | 18 (29.03) | 22 (40.74) |  |
| 2 (Bilateral) | 79 (79.00) | 67 (67.00) |  | 35 (92.11) | 39 (84.78) |  | 44 (70.97) | 28 (51.85) |  |
| **History of previous surgery, n (%)** | | | | | | | | | |
| Sinus related surgery | 13 (13.00) | 7 (7.00) | 0.239 | 8 (21.05) | 1 (2.17) | 0.015 | 5 (8.06) | 6 (11.11) | 0.810 |
| Functional endoscopic sinus surgery | 1 (1.00) | 2 (2.00) | 1.000 | 1 (2.63) | 1 (2.17) | 1.000 | 0 (0.00) | 1 (1.85) | 0.945 |
| **History of disease, n (%)** | | | | | | | | | |
| Hypertension | 5 (5.00) | 13 (13.00) | 0.084 | 1 (2.63) | 5 (10.87) | 0.301 | 4 (6.45) | 8 (14.81) | 0.242 |
| Diabetes | 2 (2.00) | 3 (3.00) | 1.000 | 1 (2.63) | 0 (0.00) | 0.923 | 1 (1.61) | 3 (5.56) | 0.515 |
| Kidney disease | 1 (1.00) | 1 (1.00) | 1.000 | 1 (2.63) | 0 (0.00) | 0.923 | 0 (0.00) | 1 (1.85) | 0.945 |
| Hepatobiliary disease | 4 (4.00) | 4 (4.00) | 1.000 | 3 (7.89) | 2 (4.35) | 0.825 | 1 (1.61) | 2 (3.70) | 0.903 |
| Gastrointestinal disease | 1 (1.00) | 6 (6.00) | 0.124 | 1 (2.63) | 2 (4.35) | 1.000 | 0 (0.00) | 4 (7.41) | 0.095 |
| Lung disease | 7 (7.00) | 3 (3.00) | 0.330 | 5 (13.16) | 2 (4.35) | 0.290 | 2 (3.23) | 1 (1.85) | 1.000 |

**Additional file 1: Table 2. Efficacy and safety outcomes for patients with functional endoscopic sinus surgery**

| **Variables** | **Overall patients** | | | **Surgery for patients with sphenoid sinus** | | | **Surgery for patients without sphenoid sinus** | | |
| --- | --- | --- | --- | --- | --- | --- | --- | --- | --- |
|  | **IGS** | **Non-IGS** | **P-value** | **IGS** | **Non-IGS** | **P-value** | **IGS** | **Non-IGS** | **P-value** |
| **Number of patients** | 100 | 100 | - | 38 | 46 | - | 62 | 54 | - |
| **Avoid rehospitalization due to bleeding, n (%)** | 100 (100.00) | 96 (96.00) | 0.121 | 38 (100.00) | 44 (95.65) | 0.499 | 62 (100.00) | 52 (96.30) | 0.215 |
| **Avoid reoperation due to bleeding, n (%)** | 100 (100.00) | 96 (96.00) | 0.121 | 38 (100.00) | 44 (95.65) | 0.499 | 62 (100.00) | 52 (96.30) | 0.215 |
| **Avoid reoperation due to recurrence, n (%)** | 100 (100.00) | 100 (100.00) | - | 38 (100.00) | 46 (100.00) | - | 62 (100.00) | 54 (100.00) | - |
| **Procedure time, min** | | | | | | | | | |
| Mean (SD) | 99.35 (42.04) | 104.13 (38.12) | 0.401 | 113.68 (47.60) | 120.61 (33.70) | 0.438 | 90.56 (35.87) | 90.09 (36.25) | 0.944 |
| Median [IQR] | 100.00  [75.00, 125.00] | 100.00  [74.25, 130.00] | 0.380 | 115.00  [90.00, 128.75] | 120.00  [95.00, 148.75] | 0.125 | 95.00  [61.25, 113.75] | 80.00  [65.00, 119.50] | 0.731 |
| **Length of stay, day** | | | | | | | | | |
| Mean (SD) | 5.95 (1.02) | 5.43 (2.21) | 0.034 | 5.92 (1.08) | 5.72 (2.33) | 0.620 | 5.97 (0.99) | 5.19 (2.10) | 0.010 |
| Median [IQR] | 6.00  [5.00, 6.00] | 5.00  [4.00, 6.00] | <0.01 | 6.00  [5.25, 6.00] | 5.00  [4.00, 6.00] | 0.035 | 6.00  [5.00, 6.00] | 5.00  [4.00, 6.00] | <0.001 |
| **Length of stay due to bleeding, day** | **n=0** | **n=4** |  | **n=0** | **n=2** |  | **n=0** | **n=2** |  |
| Mean (SD) | - | 4.00 (2.58) | - | - | 6.00 (1.41) | - | - | 2.00 (1.41) | - |
| Median [IQR] | - | 4.00  [2.00, 6.00] |  | - | 6.00  [5.00, 7.00] |  | - | 2.00  [1.00, 3.00] |  |
| **Length of stay after procedure, day** | | | | | | | | | |
| Mean (SD) | 3.88 (0.81) | 3.05 (0.89) | <0.001 | 3.79 (0.93) | 3.24 (0.90) | 0.007 | 3.94 (0.72) | 2.89 (0.86) | <0.001 |
| Median [IQR] | 4.00  [3.00, 4.00] | 3.00  [2.00, 4.00] | <0.001 | 4.00  [3.25, 4.00] | 3.00  [3.00, 4.00] | 0.001 | 4.00  [3.00, 4.00] | 3.00  [2.00, 3.00] | <0.001 |
| **Blood transfusion, n (%)** | 0 (0.00) | 0 (0.00) | - | 0 (0.00) | 0 (0.00) | - | 0 (0.00) | 0 (0.00) | - |
| **Serious adverse event, n (%)** | 0 (0.00) | 4 (4.00) | 0.121 | 0 (0.00) | 2 (4.35) | 0.499 | 0 (0.00) | 2 (3.70) | 0.215 |
| Perioperative bleeding, n (%) | 0 (0.00) | 0 (0.00) | - | 0 (0.00) | 0 (0.00) | - | 0 (0.00) | 0 (0.00) | - |
| Orbital injury, n (%) | 0 (0.00) | 0 (0.00) | - | 0 (0.00) | 0 (0.00) | - | 0 (0.00) | 0 (0.00) | - |
| Intracranial injury, N (%) | 0 (0.00) | 0 (0.00) | - | 0 (0.00) | 0 (0.00) | - | 0 (0.00) | 0 (0.00) | - |
| Bleeding after discharge from hospital, n (%) | 0 (0.00) | 4 (4.00) | 0.121 | 0 (0.00) | 2 (4.35) | 0.499 | 0 (0.00) | 2 (3.70) | 0.215 |

**Additional file 1: Table 3. Healthcare costs for all patients with functional endoscopic sinus surgery**

| **Variables** | **Overall patients** | | |
| --- | --- | --- | --- |
|  | **IGS** | **Non-IGS** | **P-value** |
| **Number of patients** | 100 | 100 |  |
| **Total costs** **(Including follow-up inpatient costs due to bleeding)** | | | |
| Mean (SD) | 17,391.51 (2,397.70) | 17,742.41 (3498.82) | 0.204 |
| Median [IQR] | 17,447.92 [15,226.44, 19,103.97] | 18,049.26 [15,427.10, 19,851.24] | 0.298 |
| **Medical service costs** |  |  |  |
| Mean (SD) | 421.86 (120.72) | 453.44 (237.24) | 0.237 |
| Median [IQR] | 360.00 [360.00, 488.80] | 385.00 [300.00, 539.20] | 0.913 |
| **Diagnosis costs** |  |  |  |
| Mean (SD) | 1,987.98 (548.64) | 2,256.84 (1,047.65) | 0.024 |
| Median [IQR] | 1,979.50 [1,601.50, 2,370.20] | 2,122.00 [1,814.95, 2,427.80] | 0.075 |
| **Procedure-related medical costs** | | | |
| Mean (SD) | 11,446.99 (1,806.33) | 10,160.87 (2,379.05) | <0.001 |
| Median [IQR] | 11,645.00 [9,973.28, 12,626.07] | 10,641.50 [8,430, 12,028.70] | <0.001 |
| **Procedure-related consumables costs** | | | |
| Mean (SD) | 1,588.19 (500.08) | 1,812.30 (693.80) | 0.009 |
| Median [IQR] | 1,497.96 [1,263.63, 1,810.83] | 1,742.22 [1,495.88, 2,057.89] | 0.001 |
| **Drug costs** | | | |
| Mean (SD) | 1,580.26 (415.61) | 2,344.27 (573.31) | <0.001 |
| Median [IQR] | 1,574.27 [1,279.59, 1,861.63] | 2,324.01 [2,009.34, 2,685.82] | <0.001 |
| **Antibacterial drugs in the drug costs** | | | |
| Mean (SD) | 198.85 (366.30) | 487.57 (193.02) | <0.001 |
| Median [IQR] | 49.98 [49.98, 49.98] | 465.24 [349.97, 591.44] | <0.001 |
| **Hospitalization-related treatment costs** | | | |
| Mean (SD) | 190.48 (44.99) | 331.49 (165.04) | <0.001 |
| Median [IQR] | 188.50 [173.88, 207.13] | 260.92 [202.85, 473.98] | <0.001 |
| **Nursing costs** |  |  |  |
| Mean (SD) | 175.75 (28.53) | 141.12 (52.03) | <0.001 |
| Median [IQR]. | 176.00 [160.00, 189.00] | 128.50 [111.00, 159.00] | <0.001 |
| **Rehabilitation costs** |  |  |  |
| Mean (SD) | 0.00 (0.00) | 37.80 (29.11) | <0.001 |
| Median [IQR] | 0.00 [0.00, 0.00] | 60.00 [0.00, 60.00] | <0.001 |
| **12-Month follow-up outpatient costs due to regular visits after FESS (n=)** | **n=91** | **n=99** |  |
| Mean (SD) | 1,362.15 (950.30) | 1,318.52 (888.76) | 0.744 |
| Median [IQR] | 1,212.95 [717.78, 1,730.46] | 1,125.20 [748.10, 1,573.33] | 0.923 |
| **12-Month follow-up inpatient costs due to bleeding (n=)** | **n=0** | **n=4** | - |
| Mean (SD) | - | 5,107.03 (2,288.41) | - |
| Median [IQR] | - | 4,236.15 [3,664.19, 5,679.00] |  |
| **12-Month follow-up inpatient costs due to recurrence (n=)** | **n=0** | **n=0** | - |
| Mean (SD) | - | - | - |
| Median [IQR] | - | - |  |

**Additional file 1: Table 4. Healthcare costs for subgroup patients with functional endoscopic sinus surgery**

| **Variables** | **Surgery for patients with sphenoid sinus** | | | **Surgery for patients without sphenoid sinus** | | |
| --- | --- | --- | --- | --- | --- | --- |
|  | **IGS** | **Non-IGS** | **P-value** | **IGS** | **Non-IGS** | **P-value** |
| **Number of patients** | 38 | 46 | - | 62 | 54 | - |
| **Total costs (Including follow-up inpatient costs due to bleeding)** | | | | | | |
| Mean (SD) | 18,764.23 (2,395.17) | 19,624.92 (3,069.37) | 0.076 | 16,550.17 (1,989.28) | 16,138.79 (3,028.48) | 0.199 |
| Median [IQR] | 19,096.88 [16,785.76, 20,397.98] | 19,548.47 [18,562.28, 20,574.16] | 0.236 | 16,506.63 [14,835.22, 18,243.81] | 16,289.18 [13,892.64, 17,691.27] | 0.327 |
| **Medical service costs** |  |  |  |  |  |  |
| Mean (SD) | 434.32 (136.29) | 453.67 (218.88) | 0.637 | 414.23 (110.58) | 453.24 (253.88) | 0.275 |
| Median [IQR] | 360.00 [360.00, 506.25] | 385.00 [302.00, 538.00] | 0.888 | 360.00 [360.00, 420.00] | 398.50 [287.00, 535.00] | 0.947 |
| **Diagnosis costs** | | | | | | |
| Mean (SD) | 2,063.22 (563.37) | 2,285.34 (693.86) | 0.116 | 1,941.86 (538.81) | 2,232.56 (1,280.67) | 0.106 |
| Median [IQR] | 2,028.00 [1,789.50, 2,356.75] | 2,156.90 [1,860.62, 2,489.80] | 0.127 | 1,905.00 [1,508.50, 2,368.25] | 2,002.90 [1,705.25, 2,367.80] | 0.379 |
| **Procedure-related medical costs** | | | | | | |
| Mean (SD) | 12,515.54 (1,837.71) | 11,682.43 (1,942.75) | 0.0471 | 10,792.08 (1,449.05) | 8,864.72 (1,909.59) | <0.001 |
| Median [IQR] | 13,057.50 [11,071.50, 13,925.64] | 12,028.70 [11,025.00, 12,820.00] | 0.0300 | 10,822.13 [9,435.63, 12,035.00] | 9,127.00 [7,205.00, 10,250.00] | <0.001 |
| **Procedure-related consumables costs** | | | | | | |
| Mean (SD) | 1,730.45 (573.27) | 1,935.08 (930.31) | 0.24 | 1,501.00 (431.50) | 1,707.72 (374.27) | 0.007 |
| Median [IQR] | 1,649.12 [1,357.10, 2,262.27] | 1,777.11 [1,629.98, 2,083.62] | 0.327 | 1,474.60 [1,248.22, 1,641.33] | 1,712.05 [1,445.12, 2,007.68] | 0.003 |
| **Drug costs** | | | | | | |
| Mean (SD) | 1,646.81 (396.99) | 2,461.53 (551.54) | <0.001 | 1,539.47 (424.61) | 2,244.38 (577.56) | <0.001 |
| Median [IQR] | 1,604.26 [1,456.36, 1,892.60] | 2,442.28 [2,119.22, 2,758.75] | <0.001 | 1,541.22 [1,231.36, 1,811.62] | 2,193.43 [1,771.55, 2,540.90] | <0.001 |
| **Antibacterial drugs in the drug costs** | | | | | | |
| Mean (SD) | 211.06 (380.43) | 512.15 (183.43) | <0.001 | 191.36 (360.31) | 466.63 (200.13) | <0.001 |
| Median [IQR] | 49.98 [49.98, 49.98] | 489.96 [394.59, 630.18] | <0.001 | 49.98 [49.98, 49.98] | 440.02 [347.38, 539.94] | <0.001 |
| **Hospitalization-related treatment costs** | | | | | | |
| Mean (SD) | 198.05 (54.37) | 359.95 (164.84) | 0.001 | 185.84 (37.87) | 307.25 (162.80) | <0.001 |
| Median [IQR] | 201.70 [175.35, 219.75] | 300.19 [218.85, 543.91] | <0.001 | 181.35 [173.98, 199.40] | 229.89 [193.33, 416.14] | <0.001 |
| **Nursing costs** | | | | | | |
| Mean (SD) | 175.84 (28.26) | 146.15 (56.81) | 0.004 | 175.69 (28.92) | 136.83 (47.71) | <0.001 |
| Median [IQR] | 176.00 [160.00, 189.00] | 131.00 [114.75, 150.75] | <0.001 | 176.00 [160.00, 189.00] | 128.50 [106.75, 164.00] | <0.001 |
| **Rehabilitation costs** | | | | | | |
| Mean (SD) | 0.00 (0.00) | 36.52 (29.61) | <0.001 | 0.00 (0.00) | 38.89 (28.92) | <0.001 |
| Median [IQR] | 0.00 [0.00, 0.00] | 60.00 [0.00, 60.00] | <0.001 | 0.00 [0.00, 0.00] | 60.00 [0.00, 60.00] | <0.001 |
| **12-Month follow-up outpatient costs due to regular visits after FESS (n=)** | **n=34** | **n=45** | - | **n=57** | **n=54** | - |
| Mean (SD) | 1,789.02 (988.55) | 1,417.73 (1,068.22) | 0.118 | 1,107.52 (835.98) | 1,235.84 (705.62) | 0.385 |
| Median [IQR] | 1,682.01 [1,201.94, 2,171.14] | 1,185.77 [793.58, 1,713.10] | 0.021 | 940.16 [613.79, 1,357.20] | 1,105.10 [720.38, 1,439.34] | 0.15 |
| **12-Month follow-up inpatient costs due to bleeding (n=)** | **n=0** | **n=2** | - | **n=0** | **n=2** | - |
| Mean (SD) | - | 6,077.32 (3,342.40) | - | - | 4,136.74 (879.17) | - |
| Median [IQR] | - | 6,077.32 [4,895.61, 7,259.04] |  | - | 4,136.74 [3,825.91, 4,447.58] | - |
| **12-Month follow-up inpatient costs due to recurrence (n=)** | **n=0** | **n=0** | - | **n=0** | **n=0** | - |
| Mean (SD) | - | - | - | - | - | - |
| Median [IQR] | - | - |  | - | - |  |
